# Upregulation of Sortilin, a Lysosomal Sorting Receptor, Corresponds with Reduced Bioavailability of Latent TGFβ in Mucolipidosis II Cells

**DOI:** 10.3390/biom10050670

**Published:** 2020-04-26

**Authors:** Jarrod W. Barnes, Megan Aarnio-Peterson, Joy Norris, Mark Haskins, Heather Flanagan-Steet, Richard Steet

**Affiliations:** 1 Division of Pulmonary, Allergy and Critical Care Medicine, University of Alabama at Birmingham, Birmingham, AL 35294, USA; barnesj5@uab.edu; 2 Greenwood Genetic Center, Greenwood, SC 29646, USAjwnorris@ggc.org (J.N.); heatherfs@ggc.org (H.F.-S.); 3 Emeritus Professor, Pathology and Medical Genetics, School of Veterinary Medicine, University of Pennsylvania, Philadelphia, PA 19104-6051, USA; mhaskins@vet.upenn.edu

**Keywords:** mucolipidosis II, sortilin, TGF-beta, lysosomes, cathepsin D

## Abstract

Mucolipidosis II (ML-II) is a lysosomal disease caused by defects in the carbohydrate-dependent sorting of soluble hydrolases to lysosomes. Altered growth factor signaling has been identified as a contributor to the phenotypes associated with ML-II and other lysosomal disorders but an understanding of how these signaling pathways are affected is still emerging. Here, we investigated transforming growth factor beta 1 (TGFβ1) signaling in the context of ML-II patient fibroblasts, observing decreased TGFβ1 signaling that was accompanied by impaired TGFβ1-dependent wound closure. We found increased intracellular latent TGFβ1 complexes, caused by reduced secretion and stable localization in detergent-resistant lysosomes. Sortilin, a sorting receptor for hydrolases and TGFβ-related cytokines, was upregulated in ML-II fibroblasts as well as *GNPTAB*-null HeLa cells, suggesting a mechanism for inappropriate lysosomal targeting of TGFβ. Co-expression of sortilin and TGFβ in HeLa cells resulted in reduced TGFβ1 secretion. Elevated sortilin levels correlated with normal levels of cathepsin D in ML-II cells, consistent with a compensatory role for this receptor in lysosomal hydrolase targeting. Collectively, these data support a model whereby sortilin upregulation in cells with lysosomal storage maintains hydrolase sorting but suppresses TGFβ1 secretion through increased lysosomal delivery. These findings highlight an unexpected link between impaired lysosomal sorting and altered growth factor bioavailability.

## 1. Introduction

The lysosomal disease mucolipidosis type II (ML-II) is caused by a defect in the biosynthesis of mannose 6-phosphate (Man-6-P) residues, the carbohydrate-based tag responsible for lysosomal targeting of acid hydrolases [1,2]. Loss of this residue results in hypersecretion of several enzymes into the extracellular space and hydrolase-deficient lysosomes. Mutations in the *GNPTAB* gene, which encodes two of the three subunits of the GlcNAc-1-phosphotransferase enzyme, lead to ML-II. The severe, multisystem manifestations of this disorder include pronounced cartilage and bone defects, many of which are noted at birth [3,4,5]. Patients with ML-II exhibit short stature, craniofacial defects and osteoporosis, which have been attributed to impaired development and function of mesenchymal cell types. An attenuated form of ML-II, referred to as ML-IIIα/β, is characterized by partial loss of mannose phosphorylation, later onset of disease symptoms and clinically distinct skeletal phenotypes [4,6,7]. The bone and cartilage findings in ML-II and ML-IIIα/β human patients are mirrored, albeit with notable differences in severity, within animal models of the disease. Phenotypic analysis of both feline and murine ML-II models has revealed changes in the cellular organization of growth plates, abnormal chondrocyte morphology and reduced endochondral ossification [8,9,10,11,12,13]. Moreover, defective chondrocyte development and excessive deposition of type II collagen were observed in a zebrafish model for ML-II [14]. These phenotypes were subsequently linked to an imbalance in TGFβ/BMP signaling caused by the inappropriate activity of the cysteine protease cathepsin K [15,16,17]. Notably, these phenotypes occur in the absence of any detectable lysosomal storage in the developing embryos [14].

Collectively, the complex nature of ML-II patient phenotypes suggests that the alterations in cell behavior and development of different tissues likely involve the dysregulation of multiple growth factor signaling pathways. Indeed, the clinical manifestations of ML-II resemble many conditions with documented growth factor dysregulation, including arthritis, osteoporosis and the congenital skeletal disorders such as Camurati-Engelmann, Marfan’s disease and geleophysic dysplasia [18,19,20,21,22,23,24]. The relevance of storage-independent alterations in growth factor signaling during early development in the context of MPSII has been documented by recent studies [25,26,27]. Despite these advances, the mechanisms whereby lysosomal dysfunction impacts the key growth factor signaling pathways implicated in aberrant bone and cartilage development and homeostasis remain largely unknown.

Transforming growth factor beta 1 (TGFβ1) mediates a broad spectrum of biological processes including wound repair, angiogenesis and immunity and plays specific roles in the development of cartilage, connective tissue and bone [28,29,30,31,32,33,34,35]. TGFβ1 and its related isoforms are initially synthesized as pre-proproteins consisting of a signal peptide, the latency-associated peptide (LAP) and the mature TGFβ1 ligand [36,37]. Prior to secretion, the TGFβ1 precursor undergoes proteolytic and post-translational modification. During this processing, the LAP portion is cleaved from the TGFβ1 ligand and non-covalently re-associates with it to form the small latent complex (SLC) [38]. In some cases, the SLC also covalently attaches to one of four latent TGFβ1 binding proteins (LTBPs), generating the large latent complex (LLC) [39]. Association with LTBPs has been shown to both facilitate rapid secretion of latent TGFβ1 and target it for storage within the extracellular matrix (ECM) [40,41]. Direct interaction between the LLC and several matrix components, such as fibrillins, fibronectin and heparan sulfate mediate growth factor latency [42,43,44,45,46]. In vivo activation of ECM-stored latent TGFβ1 can occur by various mechanisms, including those governed by integrins and thrombospondin-1 [47,48,49]. Mannose phosphorylation of latent TGFβ1 has been proposed to mediate its proteolytic activation at the cell surface [50,51]. Thus, loss of this modification could directly inhibit activation. This idea has, however, been challenged by the demonstration that latent TGFβ1 is very poorly modified with Man-6-P under physiological conditions [52].

In this study, to further address the involvement of TGFβ1 signaling in ML-II pathogenesis, the biosynthesis and regulation of this growth factor was analyzed in cultured dermal fibroblasts. One of the few available human cell culture systems for this disease, these cells exhibit the classic biochemical hallmarks of ML-II (hypersecretion of hydrolases and lysosomal storage) and are able to synthesize, secrete and respond to TGFβ1. ML-II-specific decreases in TGFβ1 signaling were noted and found to be associated with impaired wound closure and accumulation of latent complexes within the lysosomal compartment. Sortilin-1, a multifunctional lysosomal sorting receptor that has been shown to mediate lysosomal delivery of TGFβ-related cytokines, was shown to be upregulated in multiple ML-II cell models. The results of this study support two distinct molecular outcomes governed by sortilin upregulation in ML-II: (i) compensatory, carbohydrate-independent lysosomal sorting of the protease cathepsin D and (ii) impaired secretion and bioavailability of latent TGFβ1 complexes due to inappropriate delivery to this same compartment. To our knowledge, this discovery represents the first example of increased sortilin expression in the context of an inherited lysosomal storage disorder. Implications for the impaired bioavailability of latent TGFβ1 and other sortilin ligands towards ML-II pathogenesis are discussed.

## 2. Materials and Methods

Cell lines and reagents—Human control fibroblasts (CRL-1509), ML-III (GM-03391) and ML-II (GM-01586) skin fibroblasts were obtained from Coriell (Camden, NJ) and cultured in Dulbecco’s modified Eagle’s medium (DMEM) containing 18% fetal bovine serum (FBS) supplemented with 100 μg/mL penicillin in addition to streptomycin and maintained in a humidified 5% CO_2_ atmosphere. GM-01586 is homozygous for a 2-bp deletion in exon 19 of the GNPTAB gene [3665_3666delTC] resulting in a frameshift and truncation of the protein in the beta subunit [L1168fsX1172]. This cell line has been shown to have <0.1% residual GlcNAc-1-phosphotransferase activity. GM-03391 is homozygous for a 1-bp deletion at nucleotide 445 of the GNPTG gene [445delG]. Synovial membranes were taken from unaffected and ML-II feline littermates raised at the School of Veterinary Medicine at the University of Pennsylvania, under NIH and USDA guidelines for the care and use of animals in research. Fibroblast-like synoviocytes were isolated from the membranes using collagenase 1A (Sigma St. Louis, MO, USA) digestion. All primary cell lines were grown in RPMI media supplemented with 10% FBS with the addition of 100 μg/mL streptomycin and penicillin and maintained in humidified 5% CO_2_ atmosphere. HeLa cells were maintained in DMEM containing 10% FBS supplemented with penicillin and streptomycin and maintained as described above. Goat antisera against human LAP and monoclonal antibody against human LTBP was purchased from R & D systems (USA), while the monoclonal antibody γ-tubulin was obtained from Sigma. The anti-human LT-1 (LAP) rabbit polyclonal antibody was a generous gift from Dr. Kohei Miyazono (University of Tokyo, Tokyo, Japan). Rabbit antisera against human phospho-Smad 2 and monoclonal anti-human Smad 2/3 were purchased from Millipore (Billirica, MA, USA), whereas mouse anti-human sortilin-1 and GS28 were obtained from BD Transduction Labs (San Jose, CA, USA). Rabbit anti-human ERp29 and the protease inhibitor cocktail were obtained from Thermo Scientific (Rockford, IL) and Sigma (St. Louis, MO, USA), respectively. The LAMP-2 mouse monoclonal antibody was obtained from the Developmental Studies Hybridoma Bank (Iowa City, IA, USA). Rabbit anti-human cathepsin D antibody was a kind gift from Dr. Stuart Kornfeld (Washington University in Saint Louis). Recombinant TGFβ1 ligand was purchased from PeproTech Inc (Rocky Hill, NJ). TGFβ1 mink lung epithelial cells (T-MLECs) stably transfected with a luciferase gene driven by a TGFβ-responsive plasminogen activator inhibitor-1 promoter sequence was a kind gift from D. Rifkin (New York University Langone Medical Center, New York, NY, USA).

**Plasmids and constructs**—Human latent TGFβ1 was synthesized from GeneArt (Invitrogen, Carlsbad, CA, USA). The cysteine at position 33 within the TGFβ1 sequence was mutated to a serine to prevent to formation of the LLC in transfected HeLa cells to ensure the sortilin-1 was interacting with the SLC [53]. Human sortilin-1 I.M.A.G.E. clone (clone I.D. 4123836) was obtained from Open Biosystems (Thermo Scientific, Waltham, MA, USA) and was subcloned with the addition of flanking restriction digest sites (5’- Xho1 and 3’- Xba1) and ligated into pcDNA 3.1 expression vector as previously described. The glycopepsinogen in pcDNA 3.1 was kindly provided by Dr. Stuart Kornfeld (Washington University in St. Louis).

**RT-PCR analysis of transcript abundance**—RNA was extracted from pellets of ~10^6^ trypsinized control and ML-II cells using Trizol reagent (Life Technologies, Grand Island, NY, USA). RNA (500 ng) was reverse transcribed using the iScript cDNA synthesis kit (BioRad, Hercules, CA, USA). Primers used for PCR amplification (35 cycles; melting temperature: 98 °C, annealing temperature: 59 °C, extension temperature: 72 °C) were as follows—human sortilin-1 5’ TGGGTTTGGCACAATCTTTACC 3′; 5′CCACAATGATGCCTCCAGAATC 3′ and RPL4 5′ CAGAGCTGTGGCTCGAATTCC 3′; 5′ AGTTACTCTTGAGGGAAGCGGC 3′. To ensure resulting PCR products represented RNA and were not due to genomic DNA contamination, all primers were designed to exon sequences flanking an intron. RPL4 was used as a normalizing control.

**Preparation of SDS whole cell lysates, DOC (deoxycholate)-soluble, DOC-insoluble fractions and media collection**—In preparation for western blot analysis, human and feline fibroblasts were plated at equal density (2.5 × 10^5^ cells/mL), cultured for times indicated and collected by scraping with rubber policeman. For SDS whole cell lysate preparations, cell pellets were treated with 1% SDS in PBS 7.4, plus protease inhibitors followed by brief sonication and centrifugation. These cell preparations result in full solubility of cellular components. DOC-soluble and insoluble fractions were prepared as described [40]. The insoluble fraction was solubilized in SDS containing buffer (3 % SDS, 15% glycerol and 75 mM Tris pH 6.5). Both SDS whole cell and DOC preparations were normalized to total protein using micro BCA protein assay (Pierce/Thermo Scientific, Rockford, IL, USA) and prepared for SDS-PAGE and Western blot analysis as described [54]. As a loading control for SDS whole cell preps, nitrocellulose membranes were lightly stripped and re-probed for γ-tubulin. For the additional collection of conditioned media, cells were washed twice with PBS and incubated in 1 mL of serum free DMEM overnight, 24 h before collection. Media was then concentrated from 1 mL to 100 μL, in the presence of protease inhibitors, using Centricon 10 tubes (Milllipore, Billirica, MA, USA) and was assayed for equivalent loading concentration using microBCA protein assay kit (Pierce, Rockford, IL, USA).

**Wound healing assays**—Control and ML-II cells were plated and grown to confluency followed by washing with PBS and culturing cells overnight in 0.1% FBS supplement DMEM. After serum starvation, cells were scratched with a pipette tip and washed extensively to remove cell debris. Fresh culture media containing 2% FBS was added to cultures and incubated for 24 h in the presence or absence of TGFβ1 ligand (10 ng/mL). Images were prepared at 0 and 24 h post scarring using a 10x Plan C objective attached to an Olympus CX41 compound light microscope and pictures of wound closure fields were taken with a Retiga 2000R Fast 1394 camera equipped with Qcapture software v2.8.1. Measurement of wound closure was determined within several fields by defining an initial wound boundary and counting cells that migrated into the wound at the times indicated.

**Metabolic ^35^S labeling**—Pulse-chase analysis of latent TGFβ1 was performed as described [54] with modifications. Fibroblast cultures were metabolically labeled with TRAN^35^S-label (MP Biomedicals, Solon, OH, USA) in methionine- and cysteine-free DMEM (Invitrogen, USA). The media was removed and the cells were methionine deprived for 30 minutes in met/cys free media and subsequently labeled with ^35^S methionine (0.75 mCi/mL) in met/cys free media with 10% dialyzed FBS for 1 h. Media was change to DMEM supplemented with 20% FBS to initiate the chase for times indicated in each experiment. Samples were Immunoprecipitated overnight with anti-LAP antibody, incubated with protein A sepharose beads at 4 °C for 1h, washed extensively and prepared for SDS-PAGE and autoradiography detection.

**TGFβ1 mink lung luciferase activity assay**—WT and ML-II human skin fibroblasts were cultured for 3-4 days in a 60 mm dish and scraped with a rubber policeman in PBS. Cells were then pelleted by centrifugation and subsequently lysed with 1% DOC and prepared as described above. For the TGFβ1 reporter experiments, DOC-insoluble pellets were washed and broken apart using a pipette in 200 μL of DMEM (serum free). Homogenized samples were split into two 100 μL aliquots. One set was subjected to 80 °C heat activation for 10 minutes, while to other sample was not heated. After a final spin to pellet the insoluble material, media from both samples was added directly to previously plated mink lung reporter cells (1.6 × 10^5^ cells/mL) and incubated for 16 h at 37 °C. Media was then removed and cells were lysed and assayed for luciferase activity using a Luciferase Assay Kit (Promega, Madison, WI, USA).

**Percoll gradient fractionation**—Percoll gradient fractionation was performed as previously described with minor modifications [55]. After gradient formation, nine 1 mL fractions were collected from the bottom of the centrifuge tube and assayed for β-hexosaminidase activity to determine the distribution of the dense lysosomes within the collected fractions. Fractions were combined as follows—fractions 1–3 (pool I; Dense Lysosomes), fractions 4–6 (pool II; intermediate density organelles) and fractions 7–9 (pool III; Golgi, ER, endosomes and plasma membrane) based on the β-Hexosaminidase activity assays and the organelle distribution within collected Percoll gradient fractions that has been previously described [56]. Pooled fractions were precipitated with cold ethanol (100%) precipitation and subjected to SDS-PAGE and Western blot analysis.

**Co-immunoprecipitation experiments in ML-II fibroblasts**—ML-II fibroblasts were grown in a 10 cm cell culture dish to confluency. Cells were washed and collected in cold PBS followed by lysis of cells using RIPA buffer containing a protease inhibitor cocktail (Sigma, St. Louis, MO, USA) and sonication. Cell lysates were subjected to 4 °C post-nuclear centrifugation using a tabletop centrifuge. To pre-clear cell lysates, 50 μL of unblocked protein A sepharose (Pierce, Rockford, IL, USA) was added and rotated at 4 °C for 4 h. Protein A sepharose beads were removed from pre-cleared cell lysates and either antisera against sortilin-1 or LAP (LT-1) was used for the 4 °C overnight immunoprecipitation. Protein-antibody complexes were purified from cell lysates using blocked protein A sepharose followed by six washes using RIPA buffer and 2 washes with a final wash buffer containing 20 mM Tris-HCl pH 6.8. Purified protein complexes were eluded from the protein A beads by boiling in a 3% SDS buffer containing 75 mM Tris pH 6.8 and 15% glycerol and subjected to SDS-PAGE (under non-reducing conditions) and Western blot analysis. In all cases, 5% of the whole cell lysate was set aside as a Western blot input control.

**Transient transfection and latent TGFβ1 secretion assays**—HeLa cells were transfected with latent TGFβ1, glycopepsinogen, and/or sortilin-1 in Opti-MEM using Lipofectamine PLUS reagents as previously described [52]. In all co-transfection experiments, 2.5 μg of sortilin-1 and 0.3 μg of latent TGFβ1 or glycopepsinogen DNA were used. For secretion assays, transfection medium was removed after overnight incubation and cells were washed once with sterile PBS and assays were initialized by the addition of fresh Opti-MEM and incubated overnight. Media from each experiment was subjected to cold ethanol precipitation, while cells were trypsinized, collected and lysed in RIPA buffer containing a protease inhibitor cocktail followed by SDS-PAGE and Western blot analysis. To ensure equal sample loading, media aliquots were normalized total cellular protein from whole cell lysates determined by BCA protein assay (Pierce, Rockford, IL, USA). In addition, membranes were stained with Ponceau S azo dye after protein transfer or were lightly stripped and re-probed for cellular γ-tubulin to further demonstrate equal loading of media or cell samples, respectively.

**Inhibition of lysosomal function in HeLa cells with bafilomycin A1**—Latent TGFβ1 was co-transfected with sortilin-1 in HeLa cells as described above. Following the removal of the transfection medium, fresh Opti-MEM was added to the transfected cells and incubated in the presence or absence of bafilomycin A1 (10 nM) for 24 h. Cells were collected and subjected to BCA protein assay, SDS-PAGE and Western blot analysis as described above.

## 3. Results

**Figure 1:**
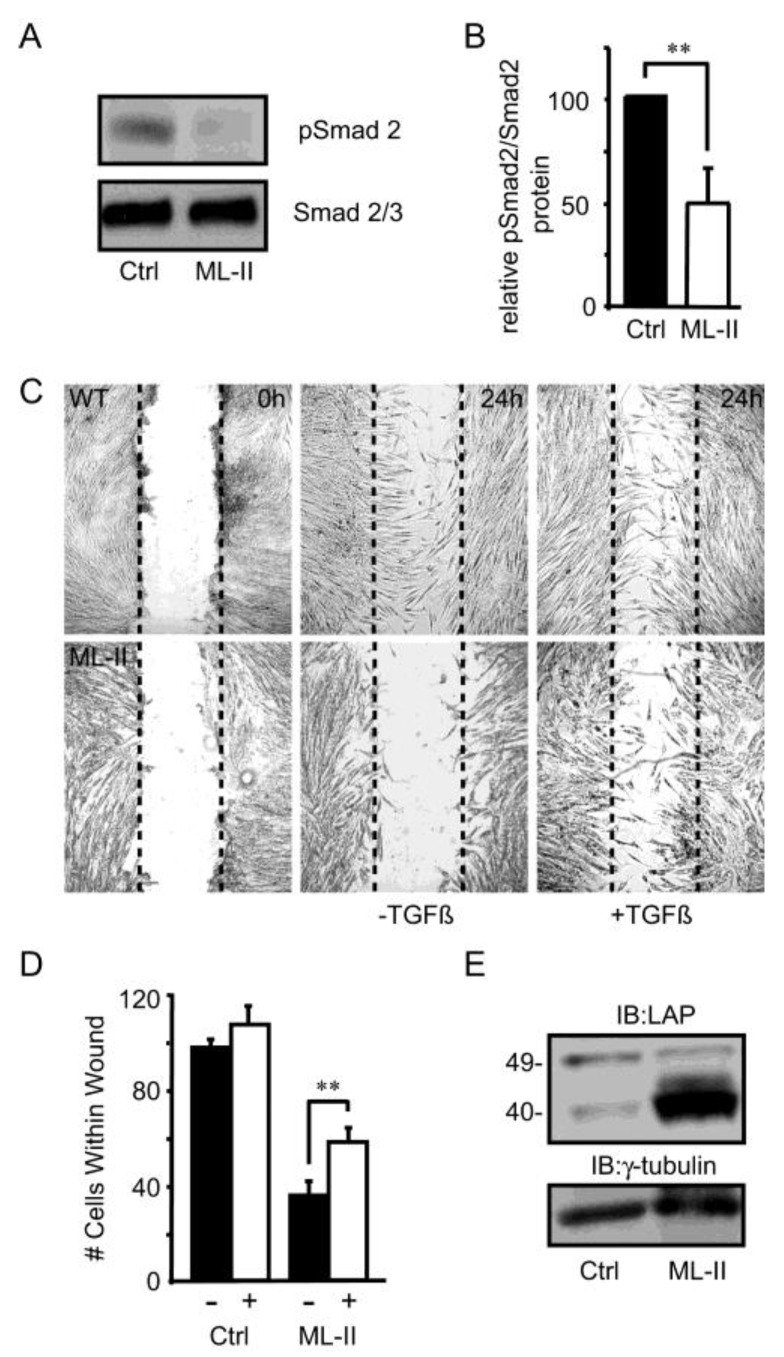
ML-II fibroblasts exhibit reduced transforming growth factor beta 1 (TGFβ1) signaling accompanied by impaired TGFβ1-dependent wound closure. (**A**) Western blot analysis of control and ML-II human fibroblast lysates using antibodies against human Smad 2/3 and phosphorylated Smad 2. (**B**) Quantification of the relative intensity of phosphorylated Smad 2 vs. Smad 2/3 protein in control and ML-II cells (n = 3), ** *p* ≤ 0.01. (**C**) Representative images of wound healing assays using control and ML-II fibroblasts in the presence and absence of 10 ng/mL TGFβ1 ligand. (**D**) Quantification of the number of cells migrating into the wounded area after 24 h (n = 3), ** *p* ≤ 0.01. (**E**) Western blot analysis of latent TGFβ1 in whole SDS lysates using an antibody against LAP. Note the pronounced increase in the level of the 40kDa LAP band in ML-II fibroblasts.

**ML-II fibroblasts exhibit reduced TGFβ1 signaling accompanied by impaired TGFβ1-dependent wound closure**—To explore whether TGFβ1 signaling is sensitive in ML-II, the level of phosphorylated Smad2 (p-Smad2), a downstream effector of TGFβ1 signaling, was investigated in control and ML-II human skin fibroblasts. The levels of p-Smad2, relative to total Smad2/3, were decreased two-fold in ML-II fibroblasts, indicating a possible reduction in canonical TGFβ1-mediated signaling (Figure 1A,B). To determine whether the decrease in phosphorylation was functionally relevant and could impact the behavior of ML-II fibroblasts, wound closure assays were performed. Wound closure following mechanical scarring is a well-established TGFβ1-dependent process, requiring the biosynthesis and remodeling of the extracellular matrix [57]. For these experiments, confluent cultures of control and ML-II fibroblasts were “wounded” and subsequently incubated in the presence or absence of TGFβ1 ligand. The extent of wound closure was assessed as the number of individual cells within the wound 24 h post-scarring (Figure 1C,D). Using this parameter, wound repair was significantly reduced in ML-II fibroblasts (63.1 ± 6.8% fewer than control fibroblasts). While addition of purified TGFβ1 did not affect this process in control cultures, it partially ameliorated the wound closure defect noted in ML-II cells. This is evidenced by an increase in the number of cells present within TGFβ1 treated ML-II wounds compared to untreated wounds (39.3 ± 13.5% fewer than control cells). These data indicate that the delayed wound closure in ML-II is at least partially TGFβ1 dependent.

In light of the positive effect that TGFβ1 addition had on ML-II wound closure, one explanation for its decreased signaling is reduced expression of the TGFβ1 precursor. Transcript analysis did not reveal any differences in TGFβ1 expression between control and ML-II samples. In contrast, Western blot analysis revealed a robust increase in steady state levels of latent TGFβ1 protein present within ML-II fibroblasts. As shown in Figure 1E, the 40-kDa band corresponding to the LAP portion of the latent complex is dramatically increased in cellular lysates derived from trypsinized ML-II cultures. These data demonstrate that the decrease in TGFβ1 signaling noted in ML-II is associated with intracellular accumulation of latent growth factor.

**Figure 2:**
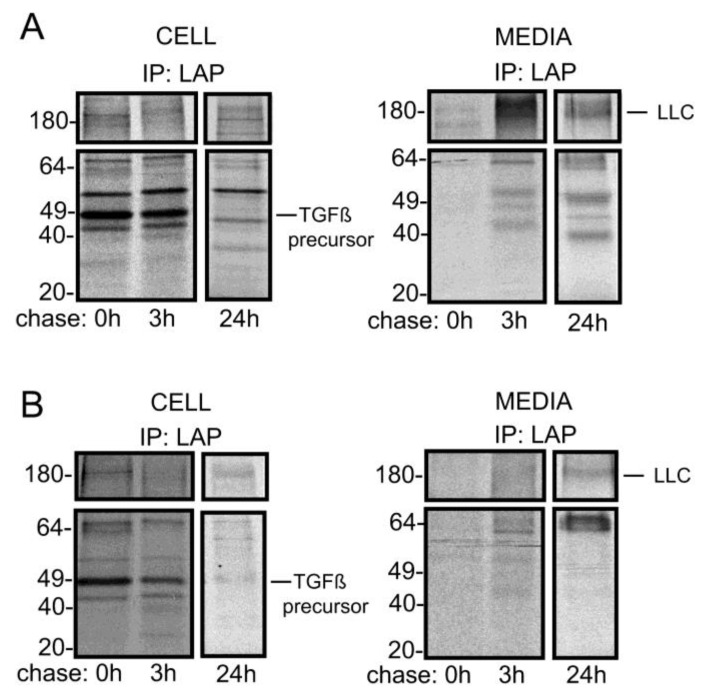
Accumulation of latent TGFβ1 in ML-II fibroblasts is associated with impaired secretion. Representative autoradiographs of ^35^S-labeled latent TGFβ1 complexes Immunoprecipitated from control (**A**) and ML-II (**B**) human fibroblast lysates and media samples at times indicated and resolved by sodium dodecyl sulfate polyacrylamide gel electrophoresis (SDS-PAGE) (n = 4). The expected migration of the TGFβ1 precursor and the large latent complex (LLC) is shown.

**The secretion of newly synthesized latent TGF-beta is decreased in ML-II fibroblasts**—To ask whether the accumulation of latent TGFβ1 within ML-II cells results from its impaired secretion, the processing and trafficking of newly synthesized growth factor was monitored using metabolic labeling experiments. As shown in Figure 2, after a 1 h pulse, ^35^S-labeled TGFβ1 precursor (49-kD) was detected within both control and ML-II fibroblasts. Analysis of media fractions (Figure 2A,B; right panel), however, revealed significant reductions in the amount of both the small latent complex (SLC) and large latent complex (LLC) secreted from ML-II cells. Increased levels of an unidentified 65-kD LAP-reactive band were also noted in the media from ML-II cultures. This 65-kDa band, along with low levels of LLC, were the only TGFβ1 related species detected within the media of ML-II cultures (Figure 2B, right panel). The identity of this band is not known but may represent a biosynthetic intermediate of latent TGFβ1. Although roughly corresponding to the molecular size expected for dimerized LAP, this protein species was completely insensitive to chemical reduction, which has been described previously [58]. Together, these data clearly show that the secretion of latent TGFβ1 is reduced in ML-II, providing a biochemical mechanism to explain the impaired signaling in these cells.

**Figure 3:**
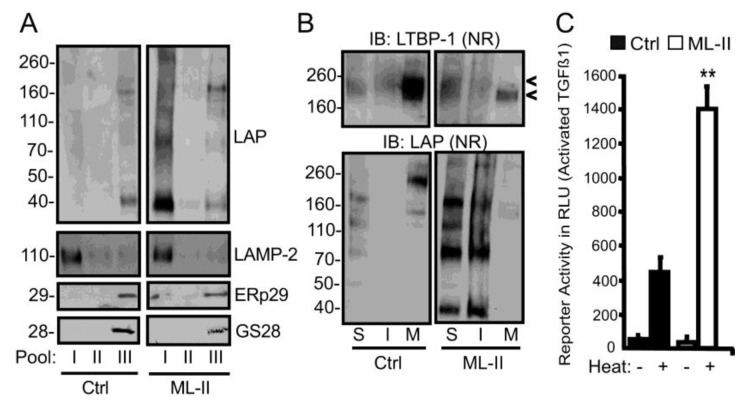
Increased intracellular latent TGFβ1 in ML-II fibroblasts is localized within dense, detergent-insoluble lysosomal fractions. (**A**) Western blot analysis of pooled Percoll gradient fractions from control and ML-II cellular lysates (n = 4). SDS-PAGE-resolved fractions were subjected to analysis using antibodies against markers for lysosomes (LAMP-2), Golgi (GS28) and ER (ERp29) as well as LAP. Pool I, dense membranes (lysosomes); pool II, intermediate density organelles; pool III, ER and Golgi membranes. (**B**) Western blots of human control and ML-II cells fractionated into deoxycholate (DOC)-soluble (S) and –insoluble (I) fractions (n = 4). Along with concentrated media (M) fractions, these pools were resolved by non-reducing (NR) SDS-PAGE and immunoblotted with antibodies against human LAP or LTBP-1. The upper and lower arrowheads indicate the position of the LLC and free LTBP-1, respectively (**C**) Luciferase assays (n = 3) of heat-treated deoxycholate-insoluble fractions that were applied to mink lung TGFβ1 reporter cells; luciferase activity was measured in relative luminescence units, ** *p* ≤ 0.01.

**Increased intracellular latent TGF-beta is localized within dense, detergent-insoluble fractions**—To identify the intracellular location of accumulating latent TGFβ1, control and ML-II cellular homogenates were fractionated using Percoll gradients and the presence of latent TGFβ1 complexes probed by Western blot (Figure 3A). Percoll gradient fractionation separates dense lysosomes and intermediate density organelles from other cellular organelles, including the endoplasmic reticulum and Golgi, providing a means to determine where intracellular latent TGFβ1 has accumulated within the secretory pathway [55,59]. The identity of individual fractions was confirmed with organelle–specific markers, including the lysosomal membrane protein LAMP-2, the ER protein ERp29 and the Golgi-associated protein GS-28. While most of the latent TGFβ1 detected in control homogenates was found in the lighter ER- and Golgi-containing pool (III), the majority of protein in ML-II homogenates was recovered in the density pool (I), which corresponds to lysosomes. Moreover, several additional high molecular weight LAP-reactive species were detected in this pool, consistent with aggregate forms of latent TGFβ1. To address whether the appearance of these higher molecular weight forms corresponds to decreased solubility of latent TGFβ1, lysates from control and ML-II fibroblast cultures grown in serum free media were treated with sodium deoxycholate (DOC) and the soluble and insoluble fractions resolved by SDS-PAGE prior to Western blot analysis. The serum free media was also collected from these cultures and analyzed. Using antisera against LAP and LTBP, the majority of latent TGFβ1 (both LLC and SLC) isolated from control fibroblasts was detected in either the media or DOC-soluble cell-associated pools (Figure 3B). In contrast, these complexes were highly enriched within both the DOC-soluble and DOC-insoluble fractions of ML-II cells, demonstrating that the increased levels of latent TGFβ1 correlate with enhanced insolubility (Figure 3B). Further, the presence of reactive bands migrating at 40 kDa, 80 kDa and 160 kDa is highly suggestive of LAP aggregation. In line with the pulse-chase analyses, ML-II media fractions were essentially devoid of both SLC and LLC. In contrast, free LTBP (~180kDa) was detected within ML-II media fractions, further suggesting that LAP is not secreted from the ML-II fibroblasts, instead leaving the free LTBP to be released from the cell alone. (Figure 3B). In the pulse/chase experiments, the use of the mild detergent to solubilize these cells, which kept any aggregated LAP insoluble, likely accounts for the absence of intracellular LAP accumulation. It is equally feasible that the pool detected in the whole SDS lysates represents protein that has stably accumulated in the lysosome with extended culture time.

**Insoluble aggregates of latent TGFβ1 in ML-II cells are capable of ligand activation**—Since SDS-PAGE conditions liberate the active ligand from latent complexes, it was not clear from the previous experiments whether the forms of LAP detected represented latent growth factor complexes yet to be “activated,” residual LAP monomers or LAP-aggregates generated following release of the active ligand. To distinguish between these possibilities, DOC-insoluble pellets from 4-day control and ML-II cultures were heat-treated to release any latent ligand and TGFβ1 activity tested using a luciferase reporter assay. A three-fold increase in TGFβ1 activity was detected in the insoluble fractions of ML-II cells following heat treatment, indicating that the accumulating latent TGFβ1 represents latent TGFβ1 complexes that are still capable of releasing active ligand (Figure 3C). Collectively, these results demonstrate that latent TGFβ1 is subject to accumulation within lysosomes in a form that is capable of activation. Furthermore, these accumulating latent complexes appear to be prone to aggregation.

**Figure 4:**
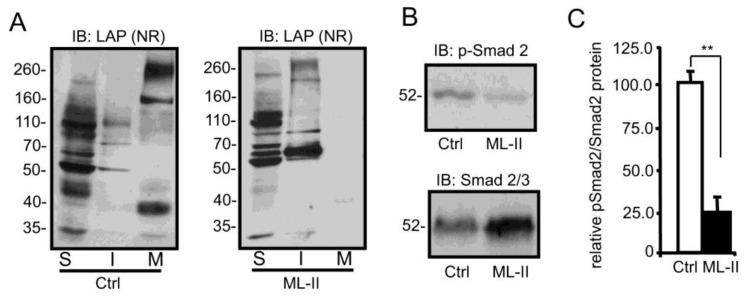
Impaired secretion and increased insolubility and of latent TGFβ1 and reduced TGFβ1 signaling, are also noted in feline ML-II fibroblast-like synoviocytes. (**A**) Western blots of feline control and ML-II cells fractionated into deoxycholate-soluble (S) and –insoluble (I) fractions (n = 3). These fractions and concentrated media (M) from cultures, were resolved by non-reducing (NR) SDS-PAGE and immunoblotted with an antibody against human LAP. (**B**) Western blot analysis of control and ML-II feline fibroblast lysates using antibodies against Smad 2/3 and phosphorylated Smad 2. (**C**) Quantification of the relative intensity of phosphorylated Smad 2 vs. Smad 2/3 protein in control and ML-II cells (n = 3), ** *p* ≤ 0.01.

**Impaired secretion and increased insolubility of latent TGFβ1 are also observed in feline fibroblast-like synoviocytes**—To establish whether this unusual biochemical profile for latent TGFβ1 is a general feature of ML-II, the localization, solubility and activity of this growth factor was investigated in feline fibroblast-like synoviocytes isolated from the synovial membranes of control and ML-II kittens. The same properties noted in human dermal fibroblasts, including reduced secretion, increased insolubility and decreased activity were observed in these cells (Figure 4). These data demonstrate that this biochemical profile is a general feature of ML-II. Interestingly, the same pattern of growth factor aggregation was not observed in the feline cells, possibly indicating that certain aspects of latent TGFβ1 processing within the secretory pathway differ between cell types or species.

**Figure 5:**
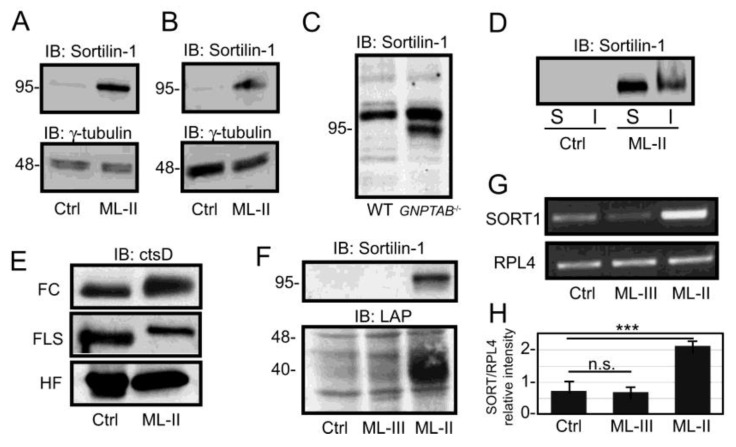
Sortilin-1 protein and transcript levels are elevated in ML-II but not ML-III, fibroblasts. Western blots for sortilin-1 in control and ML-II human fibroblasts (**A**) and feline fibroblast-like synoviocytes (**B**) using a mouse monoclonal antibody against human sortilin-1 (n = 3). (**C**) Sortilin-1 Western blot in WT and GNPTAB-/- (KO) HeLa cells (n = 2). (**D**) Sortilin-1 Western blot of DOC-soluble and –insoluble fractions of human control and ML-II fibroblasts (n = 3). (**E**) Western blot of control and ML-II feline chondrocytes (FC) and fibroblast-like synoviocytes (FLS) as well as control and ML-II human fibroblasts (HF) using a polyclonal antibody against human cathepsin D (n = 2). (**F**) Immunoblots for sortilin-1 and LAP in control, ML-III and ML-II human fibroblasts. Note the correlation of increased sortilin levels and accumulation of LAP in ML-II but not ML-III cells. (**G**) Representative reverse transcription-polymerase chain reaction (RT-PCR) analysis of sortilin-1 transcripts. Ribosomal Protein L4 (RPL4) transcript abundance is shown as a loading control. (**H**) Quantification of the intensity of SORT1 relative to RPL4 (n = 3), *** *p* ≤ 0.001.

**Sortilin-1 protein and transcript levels are increased in ML-II cells**—Having established that latent TGFβ1 is subject to lysosomal accumulation in ML-II cells, the mechanism of its delivery to this compartment was investigated. In addition to its role as a carbohydrate-independent sorting receptor for certain lysosomal hydrolases, namely cathepsins D and H and acid sphingomyelinase, sortilin has also been shown to regulate the extracellular level and lysosomal targeting of a diverse set of substrates including the TGFβ superfamily member, BMP4, BDNF, alpha-1-antitrypsin and APOB [60,61,62]. In light of this, we hypothesized that this receptor might also play a role in limiting TGFβ secretion in ML-II cells. Western blot analysis of sortilin was performed on control and ML-II fibroblast lysates. These results demonstrated that sortilin levels were substantially increased in ML-II cells relative to control cells (Figure 5A). A similar elevation in sortilin levels was also noted in the feline ML-II synoviocytes, demonstrating that higher sortilin levels are a common feature of primary ML-II cells (Figure 5B). We also demonstrated that sortilin steady-state levels were increased in CRISPR-Cas9 generated *GNPTAB*-null HeLa cells (Figure 5C), indicating that augmented sortilin expression also occurs in cells engineered to have impaired lysosomal targeting. In the human fibroblasts, elevated protein levels within DOC-soluble pools accounted for much of this increase but a detectable elevation in sortilin levels was also seen within the insoluble pool (Figure 5D).

Since sortilin has been shown to bind and play a partial role in the lysosomal delivery of cathepsin D, the possibility that ML-II cells maintain normal levels of this protease was also investigated. As shown in Figure 5E, the steady state level of cathepsin D is largely unchanged within both ML-II human fibroblasts and two different feline cell lines. Consistent with earlier reports the differences in mobility in control and ML-II cells reflect changes in glycan processing (to primarily complex-type N-glycans) that occur upon loss of mannose phosphorylation [8,63]. These findings strongly support the conclusion that this protease is capable of being retained to some extent despite the absence of Man-6-P residues and that increased sortilin-1 levels correspond to its active role in lysosomal trafficking.

Since the increased expression of this receptor may be a response to the loss of Man-6-P dependent targeting in ML-II cells, the possibility that sortilin levels were also increased in human ML-III fibroblasts was investigated. While catalytically compromised, GlcNAc-1-phosphotransferase is still expressed in ML-III; therefore, these cells retain the ability to synthesize low levels of Man-6-P residues and do not exhibit the same visible lysosomal storage and proliferation as their ML-II counterparts. The presence of residual enzyme activity in ML-III is in contrast to ML-II, where frameshift mutations yield non-functional null alleles. Interestingly, sortilin is not detectably increased in ML-III fibroblasts, suggesting a relationship between the degree of residual mannose phosphorylation and the steady state level of this alternate sorting receptor (Figure 5F). Lastly, we determined whether an increase in sortilin-1 transcripts in ML cells was associated with the elevated levels of this protein (Figure 5G). Sortilin transcripts were indeed increased in ML-II more than two-fold but not ML-III cells, indicating that these cells either actively stimulate transcription or stabilize sortilin transcripts (Figure 5H). Although some of the increase in sortilin protein levels may be a function of slower turnover or its detergent insolubility, the robust increase in sortilin-1 transcript abundance points instead to upregulation of sortilin gene expression.

**Figure 6:**
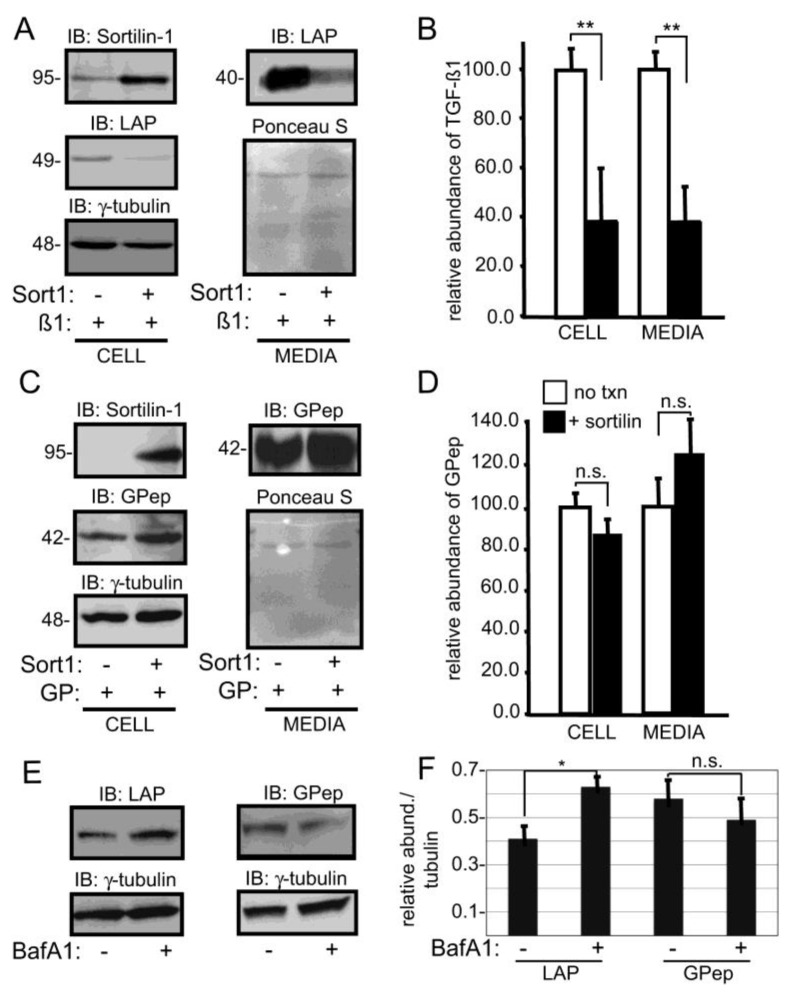
Co-expression of latent TGFβ1 and sortilin in HeLa cells reduced latent TGFβ1 secretion. (**A**) HeLa cells were transfected with DNA encoding sortilin-1 and the TGFβ1 precursor and Western blot analysis performed on cell lysates or media. Immunoblots were subsequently reprobed with antibodies specific for γ-tubulin (cell) or were stained with Ponceau S (media) as a loading control. (**B**) Relative levels of TGFβ1 in the presence and absence of exogenously expressed sortilin were quantified based on band intensity on Western blots from three separate experiments. Average intensity in cells not transfected with sortilin-1 were set to 100. (**C**,**D**) Parallel experiments and quantification in HeLa cells transfected with DNA encoding sortilin-1 and human glycopepsinogen. (**E**) HeLa cells transfected with sortilin-1 and either TGFβ1 precursor or glycopepsinogen were treated with bafilomycin A1 (BafA1) to disrupt lysosomal function and subjected to SDS-PAGE and Western blot analysis; representative images are shown. (**F**) Quantification of the intensity of LAP or glycopepsinogen relative to γ-tubulin (n = 3), * *p* ≤ 0.05, ** *p* ≤ 0.01.

**Co-expression of latent TGFβ1 and sortilin in HeLa cells reduced latent TGFβ1 secretion**—To address the link between sortilin expression and cytokine sorting, sortilin was overexpressed to determine if it could alter the secretion of latent TGFβ1 when cDNA encoding the two proteins was introduced into HeLa cells. Endogenous sortilin levels were low in HeLa cells but could be increased following transfection with sortilin cDNA, analogous to what was observed in *GNPTAB*-null HeLa cells (Figure 6A). Upon co-expression of both proteins, increased sortilin expression reduced the amount of both intracellular and extracellular latent TGFβ1, suggesting a reduction in growth factor secretion. The extent of these reductions was quantified and the results shown in Figure 6B. As a control, sortilin and glycopepsinogen, a glycosylated form of the protease pepsinogen [64], were co-expressed in HeLa cells and analyzed as before. In contrast to latent TGFβ1, neither the intracellular nor the secreted level of glycopepsinogen was sensitive to sortilin overexpression (Figure 6C,D), consistent with the ability of this sorting receptor to specifically associate with TGFβ1 and target them to the lysosome. To determine whether the decrease in intracellular levels of latent TGFβ1 (and its subsequent impaired secretion) were due to lysosomal degradation, HeLa cells co-expressing latent TGFβ1 and sortilin were treated with balifomycin, which disrupts lysosomal function and lysates from trypsinized cells were analyzed by Western blot (Figure 6E). Treatment with bafilomycin resulted in a modest increase in intracellular latent TGFβ1 levels (Figure 6F), providing further evidence that this growth factor was targeted to lysosomes and subsequently degraded when sortilin levels are increased.

**Figure 7:**
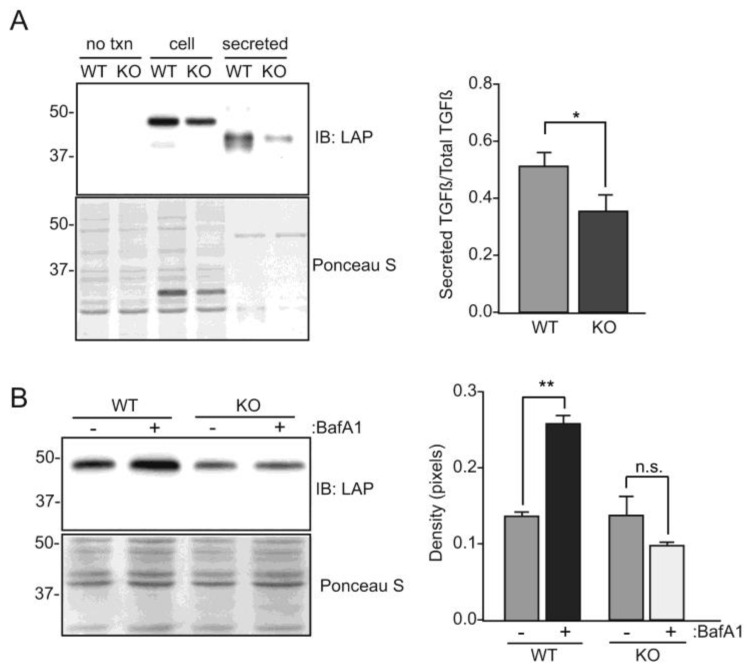
Expression of latent TGFβ1 in GNPTAB^-/-^ HeLa cells modestly lowers latent TGFβ1 secretion. (**A**) WT and GNPTAB^-/-^ HeLa cells were transfected with DNA encoding the TGFβ1 precursor and Western blot analysis performed on cell lysates or media. Immunoblots were subsequently stained with Ponceau S as a loading control. The relative amount of latent TGFβ1 to total TGFβ1 in the cell lysates and media (secreted) fractions was quantified (n = 3), * *p* ≤ 0.05. (**B**) WT and GNPTAB^-/-^ HeLa cells transfected with TGFβ1 precursor cDNA were treated with or without bafilomycin A1 and cell lysates subjected to SDS-PAGE and Western blot analysis. The abundance of latent TGFβ1 protein in cell lysates is plotted (n = 3), ** *p* ≤ 0.01.

Lastly, we assessed whether introduction of cDNA for TGFβ1 protein into the *GNPTAB*-null HeLa cells resulted in its reduced secretion. While these cells did not transfect as well as WT HeLa cells, we demonstrated that TGFβ1 secretion was lower in the *GNPTAB*-null HeLa cells (Figure 7A). Unlike the WT HeLa cells, we did not observe an increase in intracellular TGFβ1 following bafilomycin treatment in the knockout cells (Figure 7B). Rather, there was a slight reduction in the steady-state level that can be interpreted to either reflect a lack of responsiveness to the drug or that these cells lack the proteolytic capacity in their lysosomes to degrade the growth factor.

## 4. Discussion

In addition to demonstrated roles for sortilin in controlling extracellular levels of several different factors [65,66,67,68,69], numerous studies have also indicated a role for sortilin in the progression of human disorders [70,71,72]. Loss-of-function or underexpression of sortilin and its related family members has been shown to impact the pathogenic process in Alzheimer’s disease by increasing production of the Aβ peptide [73,74,75]. On the other hand, increased hepatic sortilin expression has been shown to reduce hepatic APOB secretion and increases LDL catabolism, providing a molecular basis for reduced plasma LDL-C levels and lower cardiovascular risk in humans with variations at the SORT1 locus [62,76]. The present results support a unique effect for sortilin in the context of ML-II, where the increased levels of this receptor negatively impact TGFβ1 secretion despite potential positive benefits toward hydrolase sorting. Although we did not perform experiments to directly test whether a reduction in sortilin levels in ML-II fibroblasts could reverse this phenomenon, the reciprocal relationship between its upregulation and the inappropriate lysosomal delivery of latent TGFβ1 to the lysosome is clearly reflected by the co-existence of these phenotypes in ML-II but not the closely related ML-III, fibroblasts. This hypothesis is further supported by observations that sortilin and intracellular latent TGFβ1 are present at very low levels within control fibroblasts and HeLa cells. The observation that these biochemical phenotypes are largely reproduced within early passage feline ML-II cultures is significant in that it further reinforces the disease specificity and eliminates the possibility that sortilin upregulation is simply a byproduct of fibroblasts approaching senescence. Lastly, we acknowledge the limitations in our study of only using a single control fibroblast line but note that the processing and secretion of latent TGFβ1 is highly consistent with metabolic labeling experiments from prior studies [40,41].

Latent TGFβ1 complexes accumulate in dense, detergent-resistant compartments corresponding to dense lysosomes in ML-II fibroblasts. The accumulating complexes do not appear to be subject to intracellular activation since the active TGFβ1 ligand is still capable of being released following heat treatment. Several mechanisms can be envisioned to account for this lack of activation. First, the aggregation of latent TGFβ1 complexes within detergent-resistant compartments in the ML-II cells alone likely contributes to their stability. In light of the abundant storage in ML-II cells, the aggregation and insolubility may reflect still other compensatory mechanisms utilized in these cells to manage the accumulation of storage material. Sortilin has been shown to sort prosaposin and other proteins to detergent-resistant membranes, which may explain the relative insolubility of both the receptor and ligand [77]. Second, the lysosomes in ML-II fibroblasts are generally deficient in protease activity due to the loss of Man-6-P-dependent sorting. This loss of proteolytic capacity is evidenced by the altered conversion of intermediate forms of cathepsin D to its heavy and light chain fragments within ML-II fibroblasts [63]. Since proteases are known activators of latent TGFβ1 [78], their deficiency in this compartment may spare it from efficient degradation.

Although it is unclear at this time whether increased sortilin expression is directly stimulated by the loss of Man-6-P dependent lysosomal targeting or another secondary mechanism, several observations support a direct relationship between impaired mannose phosphorylation and increased sortilin levels. First, sortilin protein and transcript levels are not increased in ML-III fibroblasts, which exhibit only partial loss of mannose phosphorylation capacity. Second, increased sortilin expression was not detected in either mucopolysaccharidosis-I or mucolipidosis-IV (mucolipin deficiency) human fibroblasts (data not shown), suggesting that elevation of this receptor is not a feature of all lysosomal storage disorders and may therefore be specific to ML-II. Sortilin has not been identified as a gene that is regulated by the transcription factor TFEB, a recently defined master regulator of lysosomal biogenesis [79,80]. This observation suggests that additional mechanisms are capable of sensing the need for Man-6-P-independent lysosomal targeting in ML-II cells. The possibility that upregulation of sortilin arises in response to either altered growth factor signaling or increased secretion of other non-lysosomal sortilin ligands also cannot be ruled out. Further analyses of the tissue-specific mechanisms that regulate sortilin expression should provide insight into these unresolved issues.

Sortilin-mediated decreases in the signaling of TGFβ1 (or any other molecules known to bind this receptor) may be relevant to the pathogenesis of ML-II and provide a novel mechanism to explain the surprising clinical distinction between the ML-II and ML-III skeletal phenotypes. The complex skeletal manifestations associated with ML-II suggest that the normal programs by which osteoblasts and chondrocytes mature are disrupted. These processes are tightly linked to the presentation and activation of TGFβ superfamily members. Our prior work in a zebrafish model for ML-II showed that increased TGFβ signaling played a central role in cartilage phenotypes associated with impaired lysosomal targeting. While this finding is seemingly contradictory to what we observed in ML-II fibroblasts, the zebrafish model lacks any signs of lysosomal storage at early developmental stages [14]. Instead the TGFβ-related phenotypes are driven by the extracellular action of cathepsin K [15,16,17]. This highlights the need to consider whether lysosomal storage is present in a particular cell type and whether sortilin expression is increased, in order to comprehensively assess any effects on TGFβ signaling. Although we did not directly assess BMP activation in the fibroblasts, a sortilin-mediated decrease in the activity of several of these growth factors in cell types that exhibit storage may contribute to the altered skeletal formation in ML-II. Indeed, sortilin upregulation occurs during osteoblast differentiation of mesenchymal stem cells, suggesting that this receptor may have a normal physiological function in bone development [81]. We have shown that another cytokine relevant to bone formation, leukemia inhibitory factor, is subject to higher extracellular levels when mannose phosphorylation and subsequent lysosomal degradation, is decreased [54].

Thus, there appear to be multiple mechanisms whereby decreased mannose phosphorylation and subsequent adaptations are capable of impacting the growth factor and cytokine signaling pathways that control bone and cartilage homeostasis. The tissue- or species-specific manner by which these factors are balanced may account for the variable nature of the phenotypes associated with the human disease as well as those defects noted within animal models of ML-II. In summary, the present findings provide a new prospective on the pathogenesis of ML-II by suggesting that increased expression of alternate hydrolase sorting receptors can negatively impact growth factor signaling in the context of impaired mannose phosphorylation.

## 5. Conclusions

The sorting receptor sortilin is specifically upregulated in ML-II cells with storage in order to compensate for the loss of carbohydrate-dependent targeting of hydrolases. This increase in sortilin expression, however, reduces the secretion of latent TGFβ by instead directing it to detergent-resistant lysosomes. These findings suggest that the enhanced expression of alternate sorting receptors can have unintended consequences on the availability and activity of growth factors and cytokines.

## Abbreviations

Man-6-P	mannose 6-phosphate;
ML-II	mucolipidosis II;
TGFβ	transforming growth factor beta;
LAP	latency-associated peptide;
SLC	small latent complex;
LLC	large latent complex;
LTBP	latent TGFβ binding protein;
LAMP	lysosomal associated membrane protein;
DOC	deoxycholate;
LDL	low density lipoprotein;
APOB	apolipoprotein B;
BDNF	brain-derived neurotrophic factor;
RPL4	ribosomal protein L4.
